# Two Paralogues as They Like It: Conserved and Divergent Evolution of Vertebrate *Gcm* Genes

**DOI:** 10.1111/ede.70057

**Published:** 2026-08-19

**Authors:** Takanori Shono, Norifumi Tatsumi, Tsutomu Miyake, Shigehiro Kuraku, Masataka Okabe

**Affiliations:** ^1^ Department of Anatomy The Jikei University School of Medicine Tokyo Japan; ^2^ Molecular Life History Laboratory, Department of Genomics and Evolutionary Biology National Institute of Genetics Mishima Japan; ^3^ Department of Genetics, School of Life Science SOKENDAI (The Graduate University for Advanced Studies) Mishima Japan

**Keywords:** comparative gene expression, gene duplication, non‐teleost vertebrates, paralogous transcription factors, pharyngeal epithelium

## Abstract

*Gcm1* and *Gcm2* are paralogous transcription factors in vertebrates that play key roles in the development of pharyngeal‐derived epithelia, yet their deployment across vertebrate lineages remains incompletely understood. While *Gcm2* shows deeply conserved pharyngeal expression across gnathostomes, *Gcm1* has been mainly characterized in mammals, where it exhibits broader expression patterns. How *Gcm1* is deployed in non‐mammalian vertebrates has remained unexplored. Here, we performed a comparative analysis of *Gcm1* expression in cartilaginous fishes, non‐teleost actinopterygians, and amphibians. RNA *in situ* hybridization revealed conserved *Gcm1* expression in gill epithelia across these taxa. Parallel analyses showed that *Gcm2* is also expressed in gill epithelia, with overlapping but distinct spatial patterns. In addition, *Gcm1* showed lineage‐specific expression in bichir embryos, including strong expression in external gills and scattered epithelial cells in the yolk‐sac membrane. In the external gills, *Gcm1*‐positive cells possess vacuole‐like cytoplasmic structures, suggesting a previously unrecognized epithelial cell population. Together, our findings indicate that *Gcm1* and *Gcm2* share ancestral expression in pharyngeal epithelia but have followed distinct evolutionary trajectories, with *Gcm1* exhibiting greater lineage‐specific diversification.

## Summary

- Comparative analyses indicate contrasting evolutionary constraints in *Gcm* paralogues. *Gcm2* shows conserved expression in pharyngeal‐derived gill epithelia, whereas *Gcm1* retains ancestral gill expression but undergoes lineage‐specific redeployment.

## Graphical abstract

Comparative overview of *Gcm1* and *Gcm2* paralogues across vertebrates. *Gcm2* is conserved in pharyngeal‐derived organs across gnathostomes, whereas *Gcm1* shows lineage‐specific diversification from an ancestral pharyngeal context, including secondary loss in teleosts, distinct expression in bichir gills and skin, and broader expression in mammals, including thymus, placenta and kidney.

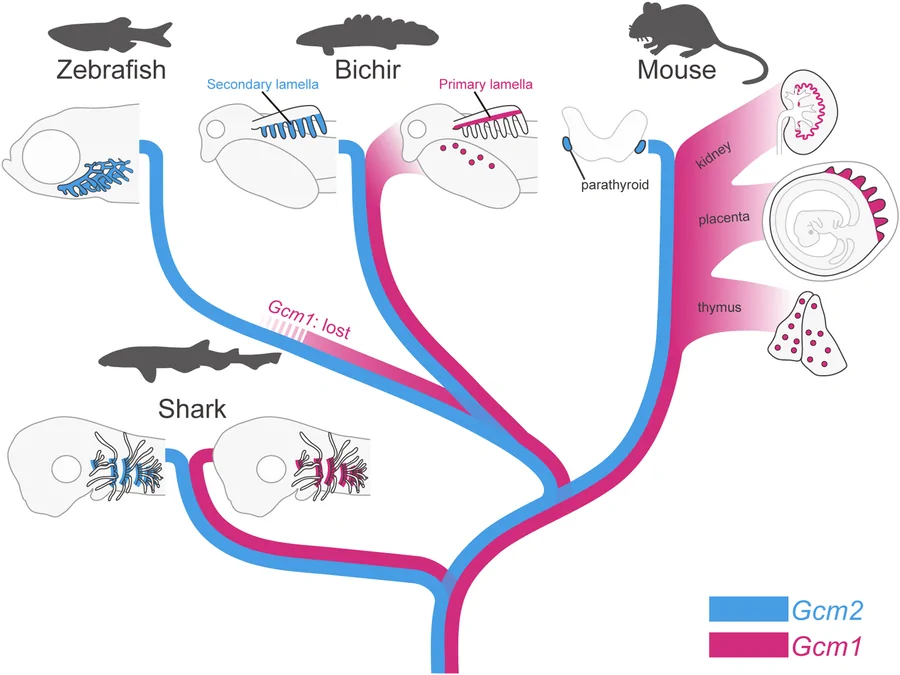

## 1 Introduction

Glial cells missing (GCM) is a zinc‐finger transcription factor that contains a specific DNA‐binding motif known as the GCM domain. It was originally identified in *Drosophila* as a binary developmental switch that specifies glial versus neural cell fate (Hosoya et al. 1995; Jones et al. 1995; Vivancos and Giangrande 1997). In vertebrates, two paralogous Gcm genes, *Gcm1* and *Gcm2* (also known as GCMa and GCMb), have been identified (Akiyama et al. 1996), providing an opportunity to examine how duplicated transcription factors diversify while retaining aspects of their ancestral developmental roles.

In mice, members of the *Gcm* gene family play essential roles in the development of non‐neural tissues. *Gcm1* is expressed in the placenta, kidney, and thymus, whereas *Gcm2* expression is restricted to the parathyroid glands (Nait–Oumesmar et al. 2000; Kim et al. 1998; Hashemolhosseini et al. 2002). Loss‐of‐function studies have demonstrated that *Gcm1* knockout mice die during embryogenesis due to defects in placental labyrinth formation, while *Gcm2* knockout mice exhibit parathyroid hypoplasia (Anson‐Cartwright et al. 2000; Günther et al. 2000; Yu et al. 2002; Yamada et al. 2019). Together, these phenotypes underscore the role of GCM transcription factors as key regulators of developmental processes and highlight how paralogous genes can acquire distinct yet developmentally critical functions.

The vertebrate pharyngeal apparatus consists of a series of arches that develop as repeated bulges along the anterior–posterior axis of the embryonic head. Through coordinated interactions among endoderm, ectoderm, mesoderm and neural crest cells, these arches undergo regional differentiation to give rise to a wide range of tissues and organs (Graham and Richardson 2012). Of the two *Gcm* paralogues, *Gcm2* plays important roles in the evolution of pharyngeal‐derived organs. In teleostean and chondrichthyan fishes such as zebrafish and small‐spotted catshark (also known as lesser‐spotted dogfish), the *Gcm2* orthologues are primarily expressed in the gills, supporting the view that gills represent the evolutionary origin of the parathyroid glands (Hanaoka et al. 2004; Hogan et al. 2004; Okabe and Graham 2004). In addition to its role in the gills, the *Gcm2* orthologues in the teleost are also expressed in the skin, where it is required for the differentiation of ionocytes (Esaki et al. 2009; Shono et al. 2011).

In contrast, the expression of *Gcm1* in cartilaginous and actinopterygian fish lineages remains largely uncharacterized. Although a *Gcm1* orthologue has been identified in the chick, its expression is restricted to the extra‐embryonic tissues (Hashemolhosseini et al. 2004). Despite the increasing availability of genomic data covering major cartilaginous and actinopterygian fish lineages, the evolutionary history of *Gcm1* remains poorly understood. The apparent absence of *Gcm1* orthologues in teleosts such as zebrafish and medaka, established model species in vertebrate genomics, has long been recognized, while its presence has been reported in cartilaginous fishes (Monroig et al. 2016). This limited understanding highlights the necessity of encompassing unexplored lineages of non‐teleost actinopterygians to examine whether *Gcm1* is present in their genomes and where it is expressed. Understanding whether *Gcm1* retained ancestral pharyngeal roles or diverged entirely in non‐mammalian lineages is crucial for reconstructing how paralogous genes evolve new developmental functions.

Within this comparative framework, polypterids, commonly known as bichir, represent an early‐diverging lineage of non‐teleost actinopterygians (Hughes et al. 2018; Patterson 1982). They retain a number of traits typical of early‐diverging lineages, including paired lungs, ganoid scales as body armor, a holoblastic egg, and dorsally positioned spiracles in the head used for air breathing (Bartsch and Britz 1997; Kerr 1907; Graham et al. 2014). Because of their unique morphological characteristics, such as their lungs, which are functionally linked to air‐breathing, polypterids have become a valuable model for evolutionary and developmental comparative studies, especially in the context of the water‐to‐land transition (Takeuchi et al. 2009; Standen et al. 2014; Tatsumi et al. 2016; Minarik et al. 2017; Stundl et al. 2019). Moreover, polypterid embryos develop prominent external gills, which serve as their primary respiratory organs during early development and resemble larval features that are observed in lungfishes and amphibians (Kerr 1907; Diedhiou and Bartsch 2009). Furthermore, recent bioinformatic analyses have revealed the whole‐genome sequence of the Senegal bichir (*Polypterus senegalus*) (Bi et al. 2021) and reedfish (*Erpetoichthys calabaricus*), which has been published in Ensembl (fErpCal1.1, INSDC Assembly GCA_900747795.2).

To address the evolutionary process of *Gcm1*, we analyzed genome sequences across a range of vertebrate species and found that the *Gcm1* gene locus is conserved in cartilaginous fishes, non‐teleost actinopterygian fishes such as bichir and sterlet (a sturgeon), and amphibians, but appears to be lost in more derived actinopterygians, including teleosts. We next examined embryonic expression patterns of *Gcm1* in representative vertebrates, including catshark, bester (an intergeneric sturgeon hybrid), bichir, amphibian axolotl and frog, and found that *Gcm1* is consistently expressed in the gill epithelia across these species. In bichir, *Gcm1* is also expressed in the skin, indicating an additional expression domain alongside the gills. These findings provide new insight into the evolutionary conservation and diversification of *Gcm1* expression and establish a foundation for reconstructing the divergent evolutionary trajectories of vertebrate *Gcm* paralogues.

## 2 Results

### 2.1 The Conserved Syntenic Region of *Gcm* Genes Across Gnathostomes

**Figure 1:**
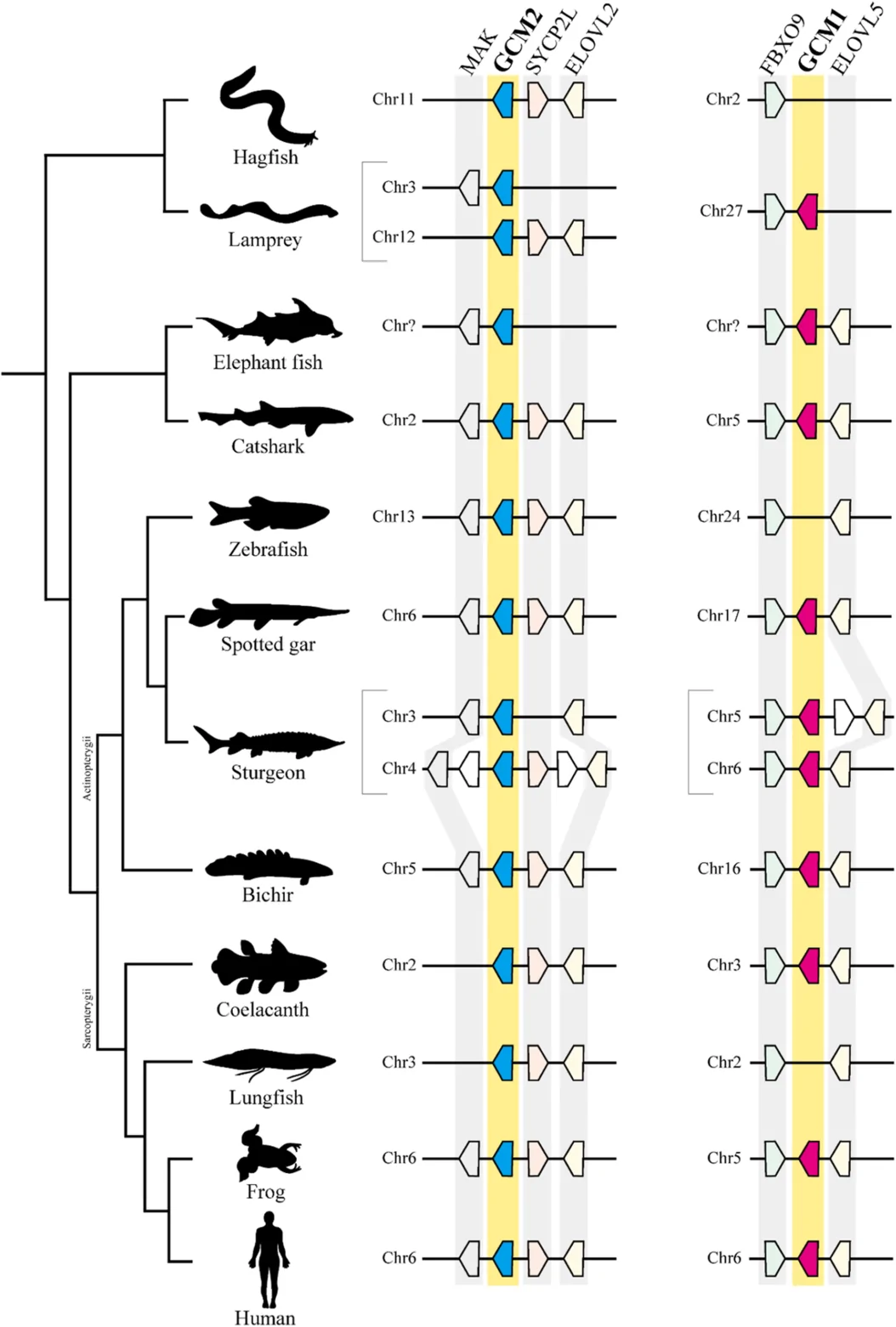
Conserved and divergent microsyntenic contexts of *Gcm2* and *Gcm1* loci across vertebrates. The left panel shows the phylogenetic relationships among the examined vertebrate species. The relationships among gnathostome lineages are based on Irisarri et al. (2017). The middle and right panels illustrate conserved syntenic regions surrounding the *Gcm2* and *Gcm1* loci, respectively. *Gcm2* orthologues are retained across all examined gnathostomes and show a highly conserved microsyntenic organization, consistent with their deeply conserved developmental roles. In contrast, *Gcm1* exhibits lineage‐specific loss, most notably in teleost fishes and lungfish (*Protopterus annectens*), despite partial conservation of the surrounding syntenic framework in other vertebrate lineages. In cyclostomes (*Eptatretus burgeri* and *Petromyzon marinus*), *Gcm*‐like loci are shown based on partially conserved synteny with gnathostome *Gcm2* and *Gcm1* regions, suggesting that a key aspect of the genomic arrangement of *Gcm* paralogue may have been established before the divergence of jawed and jawless vertebrates. [Color figure can be viewed at wileyonlinelibrary.com]

To examine the evolutionary history and genomic context of *Gcm* paralogues across vertebrates, we conducted a comparative genomic analysis focusing on the retention and syntenic organization of *Gcm2* and *Gcm1*. Our analysis revealed that *Gcm2* is retained across all examined gnathostome species, along with a highly conserved microsyntenic block consisting of *Mak*–*Gcm2*–*Sycp2l*–*Elovl2* (Figure 1 and Supporting Information S1: Figure S1). In contrast to *Gcm2*, *Gcm1* was lost in certain vertebrate lineages. Although its syntenic region was moderately conserved, a microsyntenic block consisting of *Fbxo9*–*Gcm1*–*Elovl5* was retained in certain taxa, allowing us to assess the presence or absence of *Gcm1* based on genomic comparisons (Figure 1). We confirmed that no *Gcm1* protein‐coding sequence was identified within the *Fbxo9*–*Elovl5* syntenic region in the examined teleost genomes, suggesting that this gene was lost in the teleost lineage (Supporting Information S1: Figure S2). In particular, *Gcm1* was also absent from the syntenic region of the lobe‐finned lungfish (*Protopterus annectens*) while it was retained in the coelacanth (*Latimeria chalumnae*) as well as tetrapods. In contrast, other non‐teleost actinopterygian fishes, including bichir (Cladistia), sterlet (Chondrostei), and gar (Holostei), as well as cartilaginous fishes such as catshark and elephant fish, retained the *Gcm1* gene within the conserved syntenic region of their genomes. In addition, two *Gcm2* and two *Gcm1* genes were present in the sterlet genome, suggesting that a duplication of the syntenic regions surrounding *Gcm2* and *Gcm1* occurred, probably as a result of chromosomal duplication events in Acipenseriformes (Symonová et al. 2017). These findings are consistent with an evolutionary scenario in which the *Gcm1* orthologues were lost in the common ancestor of teleosts and possibly in lungfish, while the gene and its syntenic region were preserved in non‐teleost actinopterygian fishes and cartilaginous fishes.

We also identified *Gcm*‐like genes in the lamprey (*Petromyzon marinus*) and the hagfish (*Eptatretus burgeri*), which showed partial synteny with the *Gcm2* and *Gcm1* loci in gnathostomes (Figure 1). In hagfish, the *Gcm*‐like gene was located between *Sycp2l* and *Elovl2* on chromosome 11. In lamprey, one *Gcm*‐like gene was found adjacent to *Mak* on chromosome 3, another near *Sycp2l* and *Elovl2* on chromosome 12, and a third next to *Fbxo9* on chromosome 27. These syntenic relationships suggest that the hagfish possesses a *Gcm2* homolog and that the lamprey retains two *Gcm2* and one *Gcm1* homologs. This pattern is consistent with the possibility that key aspects of the genomic arrangement of *Gcm* paralogues may have been established before the divergence of jawed and jawless vertebrates.

### 2.2 Conserved Expression of *Gcm2* in the Gills of Cartilaginous Vertebrates, Non‐Teleost Actinopterygians, and Amphibians

Because *Gcm2* expression has been reported in several vertebrate lineages (Okabe and Graham 2004; Shono et al. 2011), we first reassessed its expression in representative taxa to establish a comparative framework for lineages retaining *Gcm1*. We therefore analyzed *Gcm2* expression in the cartilaginous fish small‐spotted catshark (*Scyliorhinus canicula*), the non‐teleost actinopterygian fishes bester (a hybrid of *Huso huso* x *Acipenser ruthenus*) and bichir (*Polypterus senegalus*), and the amphibians axolotl (*Ambystoma mexicanum*) and African clawed frog (*Xenopus laevis*), using isolated RNA probes. Representative developmental stages were selected for each species based on specimen availability and the ability to clearly evaluate *Gcm* expression in developing gills; no attempt was made to establish direct developmental stage equivalence across taxa.

**Figure 2:**
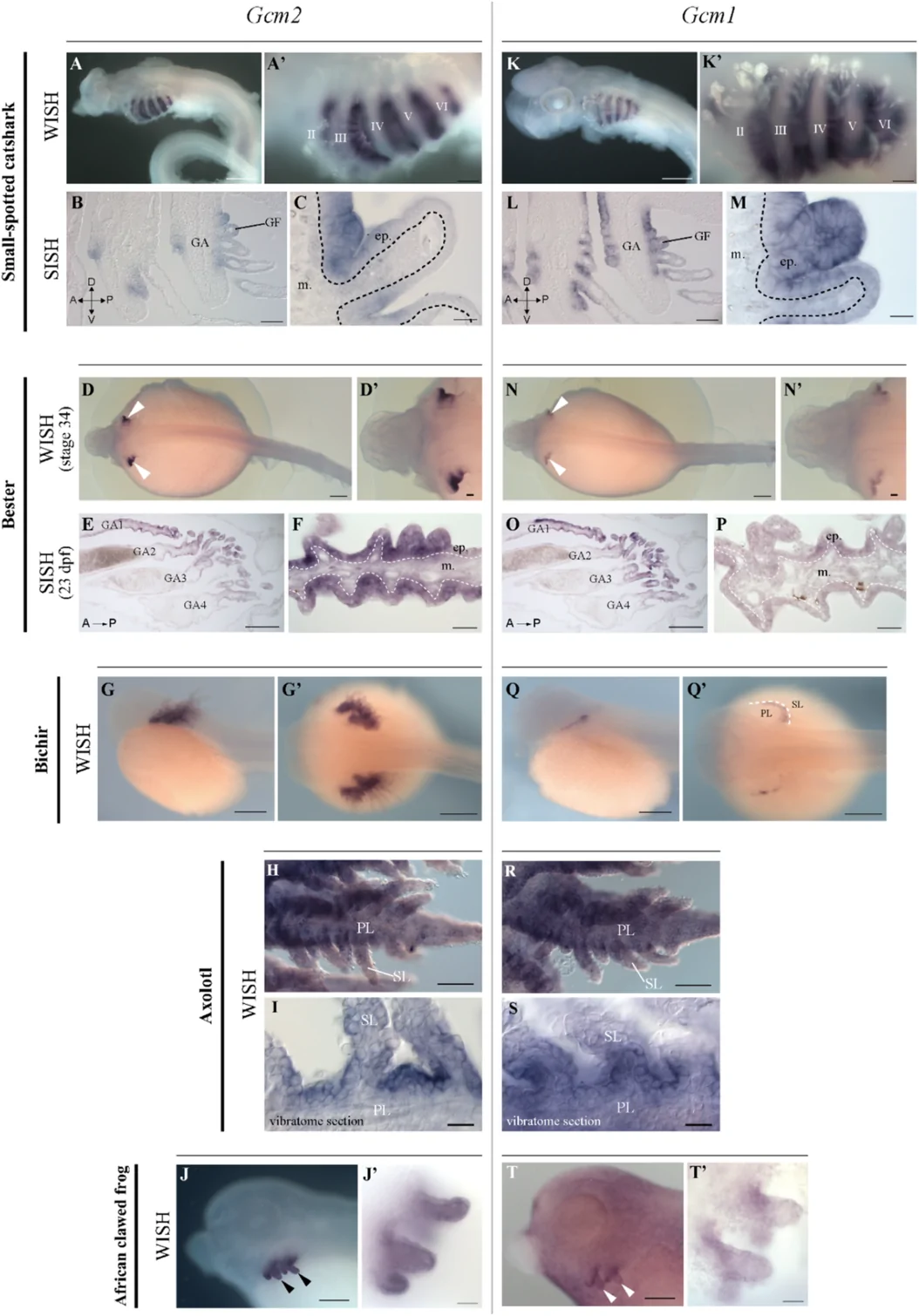
Comparative expression patterns of *Gcm2* and *Gcm1* in the gill epithelia across representative gnathostome vertebrates. RNA *in situ* hybridization was performed using species‐specific *Gcm2* and *Gcm1* probes to examine gill expression across representative gnathostome lineages. (A–C) Small‐spotted catshark embryos at stage 30 analyzed by whole‐mount *in situ* hybridization (WISH) (A, A’) and section *in situ* hybridization (SISH) (B, C). Whole‐mount and sagittal sections show *Gcm2* expression in the epithelia of secondary gill lamellae. (D–F) *Gcm2* expression in bester gills. Whole embryos at stage 34 are shown in dorsal view (D) with a magnified view of the gills (D’), in which arrowheads indicate *Gcm2* expression. Horizontal sections of gill arches in larvae at 23‐day‐post‐fertilization (dpf) (E) and higher magnification views (F) reveal epithelial *Gcm2* expression along the secondary lamellae. (G, G’) Bichir embryos at 5 dpf shown in lateral (G) and dorsal (G’) views, demonstrating *Gcm2* expression in the secondary lamellae of external gills. (H, I) Axolotl larvae at 18 dpf. (H) Dissected external gills analyzed by WISH showing *Gcm2* expression in both primary and secondary lamellae. (I) Vibratome sections prepared from the specimen shown in H confirm epithelial expression. (J, J’) African clawed frog embryos at stage 41. (J) Lateral view showing specific *Gcm2* expression in developing external gills (arrowheads). (J’) Magnified view of external gills showing strong epithelial expression of *Gcm2*. (K–M) Small‐spotted catshark embryos at stage 30 analyzed by whole‐mount *in situ* hybridization (WISH) (K, K’) and section *in situ* hybridization (SISH) (L, M). Whole‐mount and sagittal sections show *Gcm1* expression in the epithelia of secondary gill lamellae. (N–P) *Gcm1* expression in bester gills. Whole embryos at stage 34 are shown in dorsal view (N) with a magnified view of the gills (N’), in which arrowheads indicate *Gcm1* expression. Horizontal sections of gill arches in larvae at 23 dpf (O) and higher magnification views (P) reveal epithelial *Gcm1* expression along the gill lamellae. (Q, Q’) Bichir embryos at 5 dpf shown in lateral (Q) and dorsal (Q’) views, demonstrating *Gcm1* expression restricted to the basal region of the primary lamellae of external gills. (R, S) Axolotl larvae at 18 dpf. (R) Dissected external gill analyzed by WISH showing *Gcm1* expression in both primary and secondary lamellae. (S) Vibratome sections prepared from the specimen shown in R confirm epithelial expression. (T, T’) African clawed frog embryos at stage 41. (T) Lateral view showing *Gcm1* expression in developing external gills (arrowheads) and in the skin. (T’) Magnified view of the external gills showing epithelial *Gcm1* expression. Across small‐spotted catshark, bester, axolotl and African clawed frog, *Gcm1* and *Gcm2* display broadly similar epithelial expression patterns in the gills, whereas in bichir, the two paralogues show spatially distinct deployment within the external gills, with *Gcm1* localized to the primary lamellae and *Gcm2* restricted to the secondary lamellae. Paraffin sections shown in this figure were prepared at a thickness of 14 µm. Axolotl sections were prepared using a vibratome. A, anterior; P, posterior; D, dorsal; V, ventral; Pharyngeal arches 2–6 (II–VI); gill arch (GA); gill arches 1‐4 (GA1–4); gill filament (GF); primary lamella (PL); primary lamellae 1–3 (PL1–3); secondary lamellae (SL); mesenchyme (m); epithelia (ep). Scale bars: 1 mm (A, D, K, N); 500 µm (G, G’, Q, Q’); 200 µm (A’, E, H, J, K’, O, R, T); 100 µm (B, D’, L, N’); 50 µm (I, J’, S, T’); 20 µm (C, F, M, P). [Color figure can be viewed at wileyonlinelibrary.com]

In small‐spotted catshark embryos, the external gills are characterized by elongated gill lamellae protruding from the gill slits and serve as an important respiratory organ (Ballard et al. 1993). In embryos at stage 30, we observed *Gcm2* expression along the base of the external gills (Figure 2A). Section *in situ* hybridization revealed that *Gcm2* expression was largely restricted to the epithelia of the proximal region where gill lamellae elongate (Figure 2B,C). These observations are consistent with the previously reported expression of *Gcm2* in sharks and rays (Okabe and Graham 2004; Hirschberger et al. 2021; Hirschberger and Gillis 2022).

We next examined the expression of *Gcm2* in the gills of other fish species. Non‐teleost actinopterygian fishes are emerging experimental systems that represent an evolutionary bridge between teleosts and tetrapods and facilitate cross‐species comparisons. In line with an earlier study (Shono et al. 2011), *Gcm2* was also expressed in the gill regions of both bester and bichir embryos (Figure 2D–G). In stage 34 bester embryos, *Gcm2* expression was observed in the epithelia of gill arches and the developing opercular flap (Figure 2D,D’). In 23‐day‐post‐fertilization (dpf) larvae, after gill formation, *Gcm2* was detected throughout the epithelia of the secondary lamellae (Figure 2E,F). The external gills in bichir embryos consist of two segments: the primary lamella, which supports the gill structure and contains cartilaginous elements, and the secondary lamellae across which gaseous exchange occurs. In our analysis, *Gcm2* was localized to the secondary lamellae of external gills (Figure 2G,G’ and Supporting Information S1: Figure S3C,D). Furthermore, in the amphibian axolotl, which possesses three pairs of external gills of a distinct evolutionary and developmental origin from those of bichir (Stundl et al. 2019), *Gcm2* was broadly expressed in the epithelia of all external gills (Figure 2H,I). In addition, a similar broad epithelial expression of *Gcm2* was observed in the external gills of African clawed frog embryos (Figure 2J). These results suggest that *Gcm2* expression in gill epithelia is conserved among the gnathostome species examined, regardless of whether the gills are internal or external.

### 2.3 Conserved *Gcm1* Expression in Gill Epithelia Across Representative Gnathostomes

Following our analysis of *Gcm2*, we next examined *Gcm1*, revealing, for the first time, its expression in the gills of these species. In small‐spotted catshark embryos at stage 30, we observed *Gcm1* expression along the base of external gills (Figure 2K). Section *in situ* hybridization revealed that *Gcm1* expression was largely restricted to the epithelia of the proximal region where gill lamellae elongate (Figure 2L,M), whereas *Gcm2* expression extended further distally into the epithelia of the secondary gill filaments (Figure 2B,C). No clear *Gcm1* expression was observed in other tissues at this stage, suggesting gill‐specific expression of *Gcm1*.

We next examined the expression of *Gcm1* in bester and bichir and found that *Gcm1* was expressed in the gills of both species (Figure 2N–Q). In stage 34 bester embryos, *Gcm1* expression was observed in the gill arches, including the developing opercular flap (Figure 2N,N’). In bester larvae at 23 dpf, *Gcm1* was broadly expressed throughout the epithelia of the secondary lamellae, in a pattern similar to that of *Gcm2* (Figure 2E,F and Figure 2O,P). By contrast, in bichir, *Gcm1* was expressed along the primary gill lamellae (Figure 2Q,Q’), whereas *Gcm2* was localized to the secondary lamellae (Figure 2G,G’), resulting in distinct expression patterns within the same pair of external gills. Furthermore, in amphibian axolotl and African clawed frog embryos, *Gcm1* was broadly expressed in the epithelia of all external gills, exhibiting a generally similar expression pattern to that of *Gcm2* (Figure 2H–J and Figure 2R–T). The specificity of the hybridization signals was confirmed by no‐probe or sense‐probe negative controls for each species (Supporting Information S1: Figure S4).

These findings suggest that *Gcm1* expression in gill epithelia represents a conserved feature among the sampled gnathostome species that retain *Gcm1*. In bichir, however, the spatial domains of *Gcm1* and *Gcm2* in the external gills were distinct.

### 2.4 Distinct *Gcm1* Expression and a Unique Epithelial Cell Population in Bichir

**Figure 3:**
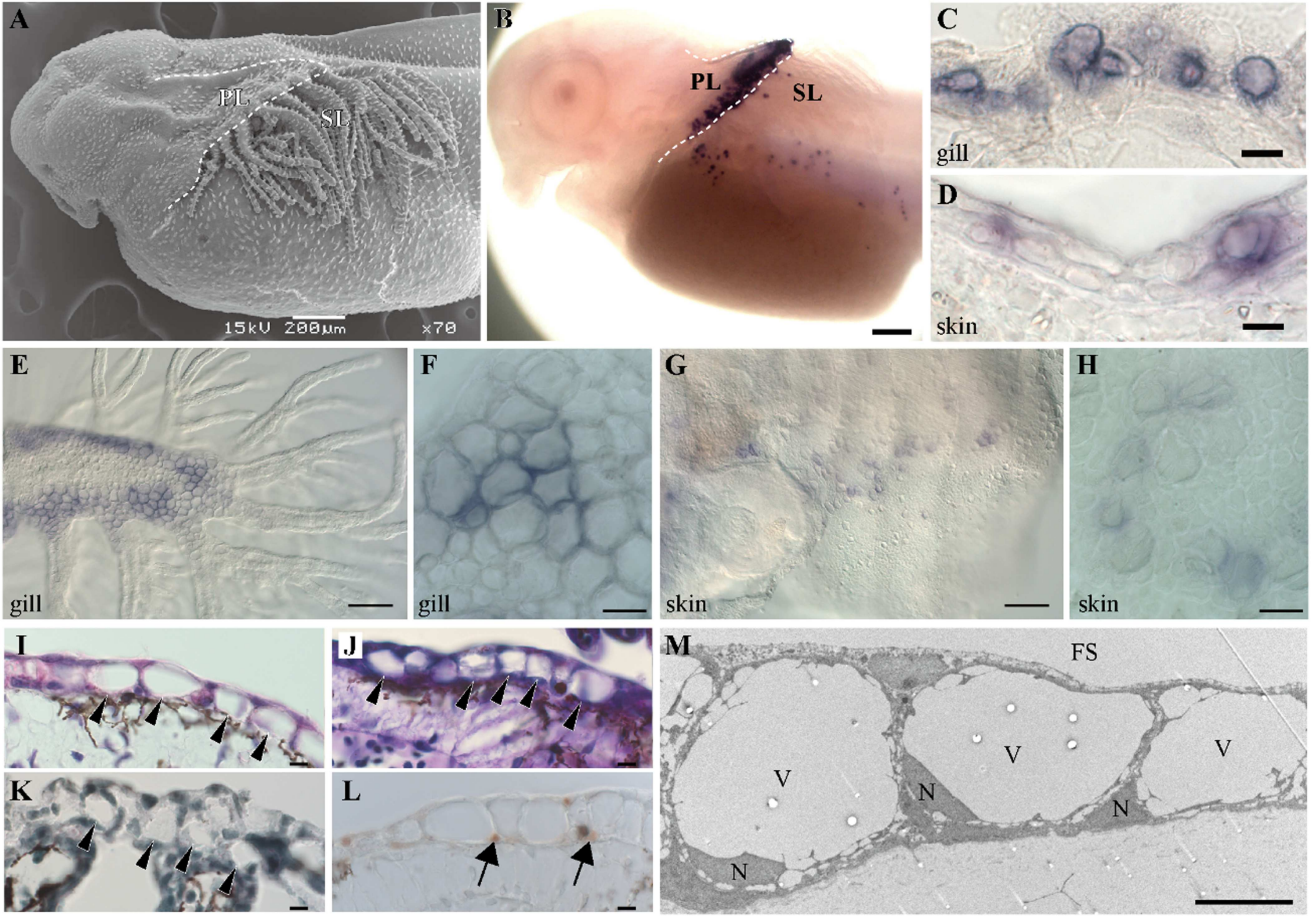
Lineage‐specific *Gcm1* expression and identification of vacuole‐like structure‐containing cells (VCCs) in bichir embryos. (A) Scanning electron micrograph of a bichir embryo at 5‐day‐post‐fertilization (dpf), showing the external gills composed of a primary lamella (PL; outlined by a white dotted line) and multiple secondary lamellae (SL). (B–H) *Gcm1* expression in bichir embryos analyzed by whole‐mount and section *in situ* hybridization. (B) Sagittal view of an embryo at 7 dpf showing *Gcm1*‐expressing cells localized to the basal region of the external gills and the skin of the yolk‐sac membrane; the primary lamella (PL) is outlined by a white dotted line and secondary lamellae (SL) are indicated. (C, D) Sagittal sections of embryos at 5 dpf showing *Gcm1* expression in the epithelia of external gills (C) and the yolk‐sac membrane (D). (E–H) Dissected external gills (E, F) and yolk‐sac membrane (G, H) from embryos at 10 dpf following whole‐mount *in situ* hybridization with a *Gcm1* probe, revealing discrete populations of *Gcm1*‐expressing epithelial cells. (I–M) Histological, immunohistochemical and ultrastructural characterization of *Gcm1*‐expressing epithelial cells in the basal region of the external gills. (I–K) Hematoxylin and eosin (I), Periodic Acid–Schiff (PAS) (J) and Sudan Black (K) staining of external gills at 10 dpf. Arrowheads indicate epithelial cells containing prominent vacuole‐like structures. These cells are negative for PAS and Sudan Black staining. (L) Immunohistochemistry for proliferating cell nuclear antigen (PCNA) in embryos at 10 dpf showing PCNA‐positive nuclei enriched within the basal epithelial layer containing vacuole‐like structure‐containing cells (VCCs) (arrows). (M) Transmission electron micrograph of the basal region of external gills at 10 dpf showing epithelial cells with a basally located nucleus (N) and large vacuole‐like structures (V) surrounded by striated cytoplasm, without detectable electron‐dense material. Paraffin sections (C, D, I, J, L) were prepared at a thickness of 14 µm, whereas Sudan Black staining (K) was performed using 10‐µm‐thick cryostat sections. FS, free surface; PL, primary lamella; SL, secondary lamellae; N, nucleus; V, vacuole‐like structure. Scale bars: 200 µm (B); 100 µm (E, G); 20 µm (C, D, F, H); 10 µm (I–M). [Color figure can be viewed at wileyonlinelibrary.com]

While *Gcm1* expression resembled that of *Gcm2* in the small‐spotted catshark, bester, axolotl and African clawed frog, it exhibited a contrasting pattern in bichir, suggesting lineage‐specific variation. Given this distinct expression pattern in bichir, we next performed a detailed analysis of *Gcm1* expression in these embryos. Scanning electron microscopy of bichir embryos revealed external gills, which are composed of a primary lamella and laterally extending secondary lamellae (Figure 3A). The first signs of *Gcm1* expression were observed in the surface cells of the primary lamellae of external gills in late embryos at 4 dpf (Supporting Information S1: Figure S3A,B). *Gcm1*‐expressing cells were largely restricted to the proximal region of the external gill arches (Supporting Information S1: Figure S3B). From 5 to 7 dpf embryos, *Gcm1* expression was also observed in scattered cells of the yolk‐sac membrane (Figure 3B). Section *in situ* hybridization revealed that epidermal cells with large vacuole‐like structures in their cytoplasm were positive for *Gcm1* mRNA (Figure 3C,D).

Further *in situ* hybridization analyses of embryos at 10 dpf showed that *Gcm1*‐expressing cells extended along the basal region of the external gills and were also present in the yolk‐sac membrane (Figure 3E–H). Isolated and magnified observations under a compound microscope revealed that *Gcm1*‐expressing cells in both external gills and yolk‐sac membrane are polygonal and pavement‐like in shape (Figure 3F,H).

Based on these distinctive morphological features, we refer to these *Gcm1*‐expressing cells as vacuole‐like structure‐containing cells (VCCs), which appear to represent a previously undescribed epithelial cell population. To investigate the contents of VCCs in the external gills, we performed Hematoxylin and Eosin staining as a standard histological stain, and additionally carried out periodic acid‐schiff (PAS) staining to detect polysaccharides and Sudan Black staining to detect lipids on paraffin sections of external gills from embryos at 10 dpf. However, no positive staining was observed in the vacuole‐like structures of VCCs (Figure 3I–K), suggesting that these cells are unlikely to correspond to PAS‐positive mucous cells or lipid‐storing adipocytes.

In mice, *Gcm1* has been implicated in the mechanisms of fibrosis and cell proliferation following ischemic injury of the kidney (Kamejima et al. 2019). To examine whether *Gcm1* plays a role in cell proliferation in bichir, we performed immunohistochemistry for the proliferation marker PCNA (proliferating cell nuclear antigen) in bichir embryos. In embryos at 10 dpf, we observed higher expression of PCNA in the external gills, specifically in the *Gcm1*‐expressing VCCs (Figure 3L). This suggests that *Gcm1* expression in VCCs may be associated with cell proliferation. We also performed transmission electron microscopy (TEM) on the external gills in bichir embryos. The results revealed no substances with visible electron density within the vacuoles of VCCs. Additionally, TEM imaging showed that VCCs are characterized by a basally located nucleus and striated cytoplasm that extends around the vacuole‐like structure (Figure 3M).

**Figure 4:**
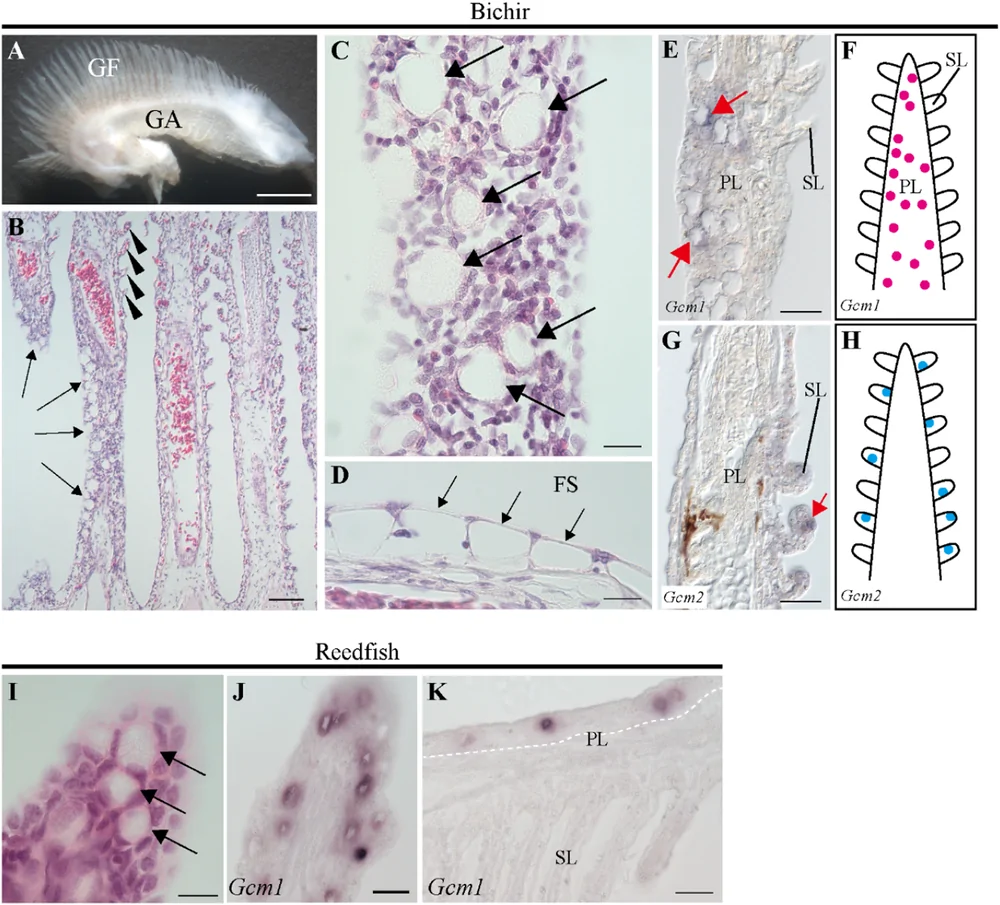
Persistence of vacuole‐like structure‐containing cells and complementary expression of *Gcm1* and *Gcm2* in adult polypterid gills. (A) Dissected gill tissue from an adult bichir showing the gill arch (GA) and gill filaments (GF). (B–D) Paraffin‐embedded sections of bichir gill filaments stained with hematoxylin and eosin. (B) Sagittal sections showing the epithelial surface of the primary lamellae. Arrows indicate vacuole‐like structure‐containing cells (VCCs) within the epithelium of the primary lamellae, whereas arrowheads indicate secondary lamellae. (C, D) Higher magnification views of the primary lamellae showing VCCs in sagittal (C) and transverse (D) sections (arrows). (E) Sagittal section of adult bichir gill analyzed by section *in situ* hybridization (SISH) with a *Gcm1* probe, showing expression in VCCs located within the primary lamellae (arrows). (F) Schematic summary of *Gcm1* expression in adult bichir gills, with magenta dots indicating *Gcm1*‐expressing cells localized to the primary lamellae. (G) Sagittal section of adult bichir gill analyzed by SISH with a *Gcm2* probe, showing expression in epithelial cells of the secondary lamellae (arrow). (H) Schematic summary of *Gcm2* expression in adult bichir gills, with blue dots indicating *Gcm2*‐expressing cells localized to the secondary lamellae. (I–K) Histological and gene expression analysis of reedfish gills. (I) Paraffin‐embedded section stained with hematoxylin and eosin showing vacuole‐like structure‐containing cells in the epithelium of the primary lamellae (arrows). (J, K) Sagittal (J) and transverse (K) sections analyzed by SISH with a *Gcm1* probe, showing epithelial *Gcm1* expression in the primary lamellae. The white dashed line indicates the boundary between the epithelium and mesenchyme of the primary lamella. Paraffin sections shown in this figure were prepared at a thickness of 14 µm. FS, free surface; GA, gill arch; GF, gill filament; PL, primary lamella; SL, secondary lamellae. Scale bars: 1 mm (A); 100 µm (B); 50 µm (E, G); 20 µm (C, D, J, K); 10 µm (I). [Color figure can be viewed at wileyonlinelibrary.com]

Our findings of *Gcm1* expression and the presence of VCCs in bichir embryos prompted us to investigate whether VCCs are also observed in adult bichir. In adult bichir, the external gills undergo regression and are eventually replaced by the internal gills and lungs as the principal respiratory organs. RT‐PCR using cDNA synthesized from RNA extracted from several organs of adult bichir showed strong expression of *Gcm1* in the internal gills, suggesting that *Gcm1* expression is enriched in adult gills (Supporting Information S1: Figure S5A). Histological staining of adult gill sections confirmed the presence of VCCs in the superficial layer of primary lamellae (Figure 4A–D). The section *in situ* hybridization also showed *Gcm1* expression in VCCs within the adult gills (Figure 4E,F). These results indicate that the expression of *Gcm1* in VCCs persists from the embryonic external gills to the adult internal gills. In addition, we observed *Gcm2* expression in the cells of the secondary lamellae, demonstrating distinct expression patterns between *Gcm1* and *Gcm2* in the adult gills (Figure 4G,H).

Lastly, we examined gill tissues from other non‐teleost actinopterygian fish species, including spotted gar (*Lepisosteus oculatus*), bester, bowfin (*Amia calva*) and reedfish (*Erpetoichthys calabaricus*) to identify the presence of VCCs. While we observed VCCs in the gills of reedfish (Figure 4I), no cellular structures analogous to VCCs were identified in the gills of spotted gar, bester or bowfin (Supporting Information S1: Figure S6). We also confirmed *Gcm1* expression in VCCs within the adult gills of reedfish (Figure 4J,K). These observations suggest that VCCs may represent a lineage‐specific epithelial population in polypterids (Cladistia), including bichir and reedfish.

## 3 Discussion

This study reveals distinct trajectories of genomic retention and expression for the two vertebrate *Gcm* paralogues across gnathostomes. It has been well established that *Gcm2* exhibits extensive genomic conservation and a highly conserved expression pattern. Our analysis indicates that the *Gcm2* gene is retained in the genomes of all examined gnathostome species, together with a largely conserved microsyntenic block (Figure 1). Moreover, *Gcm2* expression was consistently observed in the epithelia of secondary gill lamellae across the fish and amphibian species examined, revealing a strikingly conserved spatial pattern consistent with an ancestral role in pharyngeal‐derived epithelia (Figure 2).

In contrast to the highly conserved *Gcm2*, *Gcm1* exhibits lineage‐specific genomic loss and diversification in its expression patterns. Its absence in teleost genomes suggests that a gene loss event may have occurred in their common ancestor. In non‐teleost actinopterygian fishes examined in this study, such as bichir and bester—which diverged before the emergence of teleosts—*Gcm1* is retained and expressed in both gills and lineage‐derived epithelial contexts. In polypterids (bichir and reedfish), *Gcm1* is further expressed in vacuole‐like structure‐containing cells (VCCs), representing a candidate lineage‐specific epithelial population. In mammals, *Gcm1* is likewise expressed in non‐pharyngeal organs, including the placenta, kidney, and brain (Hashemolhosseini et al. 2002; Hitoshi et al. 2011). RT‐PCR in bichir and axolotl showed weak *Gcm1* expression in the skin, kidneys, reproductive organs and brain, in addition to the gills (Supporting Information S1: Figure S5). Together, these observations suggest that evolutionary constraints acting on *Gcm1* have been less stringent than those acting on *Gcm2*, potentially permitting broader diversification of its expression. Notably, BAC‐based transgenic analyses in zebrafish demonstrated that *cis*‐regulatory elements sufficient to drive *Gcm2* expression in ionocytes are contained within the *Gcm2* genomic locus, whereas gill expression could not be recapitulated by the same constructs (Shono et al. 2011). This finding suggests that *cis*‐regulatory elements responsible for ancestral gill expression may not reside in proximal promoter regions and may instead depend on distant or higher order regulatory contexts. Molecular studies in mammals further indicate differences in protein stability and transactivation potential between GCM1 and GCM2 (Tuerk et al. 2000), consistent with a model in which *Gcm1* is more permissive to regulatory and functional diversification.

**Figure 5:**
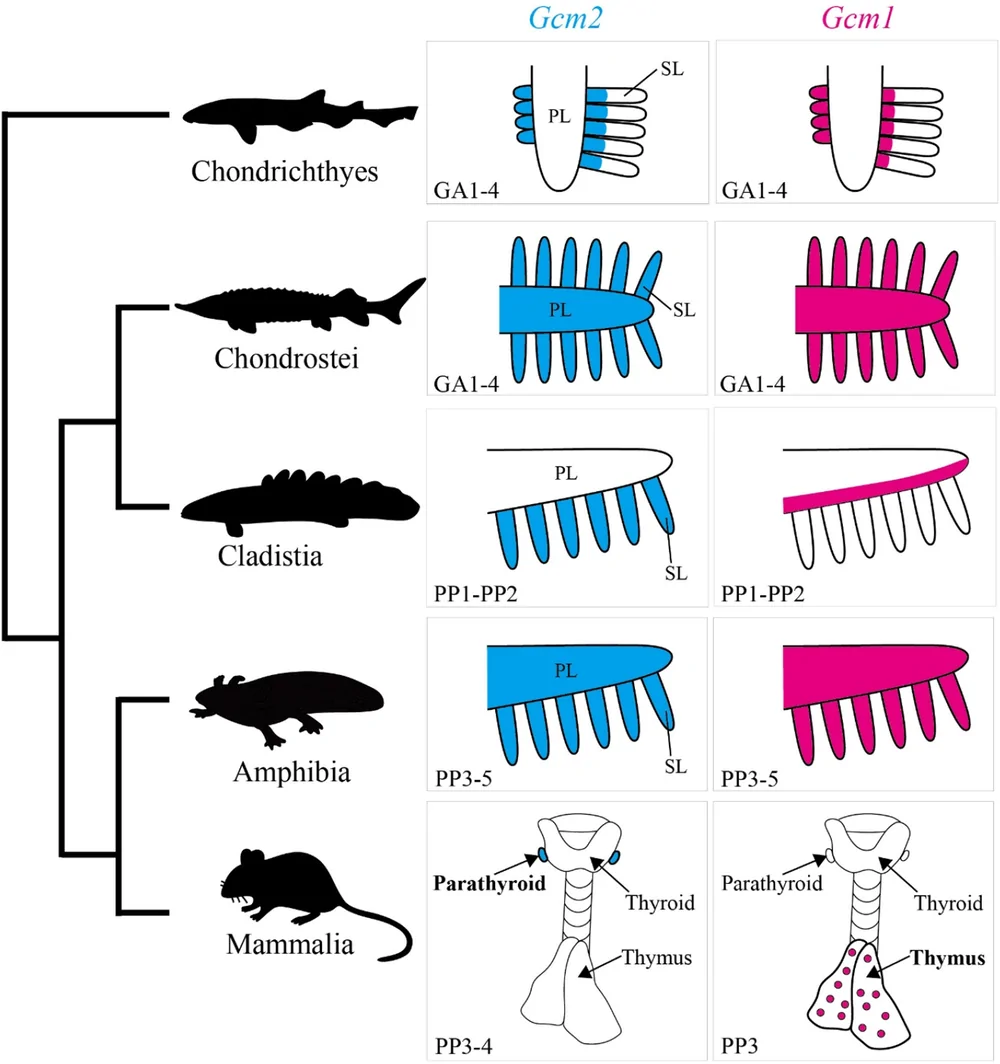
Evolutionary conservation and divergence of *Gcm2* and *Gcm1* expression across gnathostomes. Schematic summary of comparative expression patterns of *Gcm2* (blue) and *Gcm1* (magenta) across major gnathostome lineages, based on data obtained in this study and previously reported expression patterns in mammals (Nait–Oumesmar et al. 2000; Kim et al. 1998; Hashemolhosseini et al. 2002). Representative phylogenetic relationships are shown on the left. For each lineage, schematic illustrations depict the spatial deployment of *Gcm2* and *Gcm1* within pharyngeal‐derived organs. In cartilaginous fishes and non‐teleost actinopterygians, both paralogues are expressed in gill epithelia, with largely overlapping domains in chondrichthyans and chondrosteans. In polypterids, the two genes exhibit spatially distinct deployment within the external gills, with *Gcm2* localized to the secondary lamellae and *Gcm1* restricted to the primary lamellae. In amphibians, both genes are broadly expressed in gill epithelia. In mammals, *Gcm2* expression is confined to the parathyroid glands, whereas *Gcm1* is expressed in the thymus, reflecting lineage‐specific redeployment of paralogous gene functions. Numbers at the lower left of each schematic indicate the corresponding pharyngeal arch or pouch positions (GA, gill arch; PP, pharyngeal pouch). PL, primary lamella; SL, secondary lamella. [Color figure can be viewed at wileyonlinelibrary.com]

Because fish and amphibian gills, as well as the mammalian thymus (*Gcm1*‐expressing) and parathyroid glands (*Gcm2*‐expressing), all originate from the pharyngeal region, we propose that the expression of *Gcm1* and *Gcm2* has been evolutionarily conserved among pharyngeal‐derived structures across gnathostomes that have retained these genes (Figure 5). *Gcm2* was also detected in the developing opercular flap of bester embryos (Figure 2D,D’), consistent with previous observations in zebrafish (Richardson et al. 2012), suggesting that expression in the hyoid arch/opercular region may be conserved among distantly related bony fish lineages. The homology between gill‐associated pharyngeal epithelia and the mammalian parathyroid—both of which express *Gcm2*—is consistent with this continuity (Okabe and Graham 2004). By analogy, *Gcm1* expression in the thymus may reflect retention of an ancestral association with pharyngeal‐derived developmental contexts.

The similar expression patterns of *Gcm1* and *Gcm2* in the gills of the small‐spotted catshark, bester, axolotl and African clawed frog suggest that these genes may play important roles in gill development, potentially through cooperative or partially overlapping regulatory mechanisms. In contrast, mammalian *Gcm1* and *Gcm2* display spatially distinct expression patterns and regulate different downstream targets (Kamitani‐Kawamoto et al. 2011; Lo et al. 2017; Jeyarajah et al. 2022), suggesting that an evolutionarily conserved but as yet unidentified regulatory framework may underlie their coordinated expression in gills of non‐mammalian gnathostomes.

In mammals, *Gcm1* has acquired further lineage‐specific roles, most notably in the placenta, where it is required for villous development (Anson‐Cartwright et al. 2000). In humans, alterations in GCM1 activity are associated with pre‐eclampsia, underscoring its functional diversification in a non‐pharyngeal context (Baczyk et al. 2009). Together, these findings suggest that *Gcm1* has been repeatedly co‐opted into new developmental environments, acquiring diverse functions across vertebrate lineages.

In fishes and amphibians, the primary functions of gills are gas exchange and ion regulation. In teleosts, pillar cells mediate gas exchange, whereas ionocytes regulate ion homeostasis. Recent studies, particularly in zebrafish, have identified additional epithelial cell types, including pavement cells, mucous‐secreting immune‐associated cells, and oxygen‐sensitive neuroepithelial cells (Chang and Hwang 2011; Evans et al. 2005; Zachar and Jonz 2012; Pandey et al. 2021; Wilson and Laurent 2002). In contrast, the external gills and skin of bichir display epithelial features, such as the presence of ciliated cells, that differ from those described in teleosts and resemble aspects observed in amphibians. Accordingly, the epithelial cell populations of bichir do not appear to correspond directly to those characterized in teleost models, suggesting a distinct cellular organization within the gill epithelium of this lineage.

We suggest that VCCs in bichir may represent a previously uncharacterized epithelial cell population. These cells appear to differ from all known teleost epithelial cell types in morphology, ultrastructure, and marker expression. Although they share some characteristics with pavement cells (Evans et al. 2005), including a polygonal shape and apical microridges, VCCs are characterized by the presence of large vacuole‐like cytoplasmic structures, which are absent from teleost pavement cells. They also differ from mucous and club cells, as they are PAS‐negative and contain basally located nuclei. Moreover, the expression of ionocyte markers in the gill lamellae and yolk‐sac membrane did not overlap with that of *Gcm1*‐expressing cells (Supporting Information S1: Figure S7). Together, these observations suggest that VCCs are unlikely to be homologous to any previously characterized epithelial cell type in teleosts.

During embryogenesis, VCCs are PCNA‐positive, suggesting that *Gcm1* may be associated with cell proliferation in this lineage. A similar role for *Gcm1* has been reported in ischemic mouse kidneys (Kamejima et al. 2019), raising the possibility of partial functional conservation across vertebrates. Further ultrastructural and functional analyses, including targeted manipulation of *Gcm1* in bichir, will be required to clarify the developmental roles and evolutionary significance of this *Gcm1*‐expressing epithelial population.

In fish embryos, ion and gas exchange is mediated by the gills and skin, whereas in mammalian fetuses these functions are carried out by the placental villi (Evans et al. 2005; Turko et al. 2021; Burton and Fowden 2015). Notably, *Gcm1* is expressed in all of these tissues, suggesting a potential role in epithelial functions associated with fluid exchange. Its dual expression in gills and skin of bichir embryos may therefore reflect an ancestral pattern of deployment, raising the possibility that placental expression in mammals may have arisen through the redeployment of an ancestral epithelial regulatory program, originally associated with pharyngeal‐derived epithelia and associated with epithelial differentiation and transport functions.

Thus, the contrasting evolutionary trajectories of *Gcm1* and *Gcm2* illustrate a general principle of gene duplication: while one paralogue may retain ancestral characteristics, the other may become associated with different developmental contexts during evolution. This pattern is consistent with established models of duplicated gene evolution (Lynch and Conery 2000). In this sense, the evolutionary history of the *Gcm* gene pair recalls the relationship between Oliver and Orlando in Shakespeare's *As You Like It*. While the elder brother remains tied to the ancestral estate, preserving familial continuity, the younger ventures into unfamiliar environments where he explores new identities and relationships. Similarly, the conserved role of *Gcm2* may have provided developmental stability, whereas *Gcm1* became associated with a broader range of developmental contexts in different vertebrate lineages. This analogy is intended only to illustrate contrasting evolutionary trajectories rather than to imply functional divergence. By uncovering an ancestral gill‐associated role of *Gcm1* that was previously obscured by lineage‐specific gene loss, our study highlights the importance of phylogenetically informed comparisons for reconstructing hidden evolutionary histories.

## 4 Materials and Methods

### 4.1 Animals

Adult Senegal bichir (*Polypterus senegalus*), reedfish (*Erpetoichthys calabaricus*), bowfin (*Amia calva*), and axolotl (*Ambystoma mexicanum*) were purchased from Meito‐Suien Co. Ltd (Aichi, Japan), and spotted gar (*Lepisosteus oculatus*) were purchased from Paupau Aqua Garden (Ginza, Japan). The bichir, reedfish, bowfin, and spotted gar were maintained at 28°C, while the axolotl were maintained at 20°C. The fertilized eggs of bichir were raised to the desired embryonic stages in freshwater at 28°C in the laboratory. The sturgeon used for the expression analyses in this study was a hybrid of *Huso huso* x *Acipenser ruthenus* (bester). Their eggs were supplied by Fujikin Incorporated, and embryos were incubated in freshwater at 20°C. Embryos were staged according to Ballard and Needham (1964). Small‐spotted catshark embryos were sampled from fertilized eggs incubated at 14°C in Tokai University Marine Science Museum (Shizuoka, Japan) in accordance with the Husbandry Guidelines approved by the Ethics and Welfare Committee of the Japanese Association of Zoos and Aquariums and were staged according to Ballard et al. (1993). African clawed frog (*Xenopus laevis*) were purchased from Xenopus Farm (Ibaraki, Japan) and kept at 23°C. Embryos were staged according to Nieuwkoop and Faber (1994). The fertilized eggs of the African clawed frog were raised to the desired embryonic stages in freshwater at 23°C in the laboratory. All animal experiments were approved by the Animal Care and Experimentation Committee of the Jikei University School of Medicine (Nos. 2017‐066, 2017‐085, 2020‐021, 2022‐078, S‐L‐102C1) and the Institutional Animal Care and Use Committee at National Institute of Genetics (Approval ID: R5‐14 and R6‐13) and were performed in accordance with the approved guidelines, with efforts to minimize animal suffering.

### 4.2 Synteny

We retrieved the genomic database of NCBI Genome Data Viewer, Ensembl (v112), and Genomicus (v110.01) with the aim of finding synteny blocks flanking the *Gcm2* and *Gcm1* orthologues of vertebrate species.

### 4.3 Histological Analysis

Samples for histological staining were fixed in 4% paraformaldehyde in phosphate‐buffered saline) at 4°C overnight and dehydrated in a graded series of methanol. For paraffin embedding, the dehydrated samples were incubated 20 min with isopropanol and two times for 30 min with xylene. YAMATO REM‐710 microtome was used to section paraffin‐embedded samples at 14 µm. Slides were stained with several stains, including Mayer's Hematoxylin (Muto Pure Chemicals) for 10 min and Schiff Reagent (Muto Pure Chemicals) for 10 min for each purpose of the staining. Stained slides were washed with double distilled water and dehydrated through graded ethanol solutions, cleared in xylene, and coverslipped with Malinol (Muto Pure Chemicals). For Sudan Black staining, fixed samples were frozen with the aid of Tissue‐Tek OCT compound (Sakura Finetek) and 10 µm thick cryostat sections were cut on a Cryostat Leica CM3050S. The sections were stained with Sudan Black (Wako) at 37°C for 5 min and then with Nuclear Fast Red (TCI Chemicals) for 5 min. Stained slides were then washed with double distilled water and coverslipped with glycerol. Finally, mounted slides were photographed by using a Zeiss Axio imager D1BX51 compound microscope equipped with an Axiocam 512 color camera.

### 4.4 Immunohistochemistry

Bichir embryos for immunohistochemistry were fixed in 4% paraformaldehyde in PBS at 4°C overnight and dehydrated in a graded series of methanol. The dehydrated samples were incubated 20 min with isopropanol and two times for 30 min with Xylene. YAMATO REM‐710 microtome was used to section paraffin‐embedded samples at 14 µm. The paraffin sections were de‐paraffinized, rehydrated, and heated with 10 mM citric acid in double distilled water (pH 6). Slides were washed with PBST (0.01 M phosphate buffered saline, 0.01% Tween‐20) and immunoreacted overnight at 4°C with an antibody as desired experiments (rabbit anti ‐PCNA (ab18197, 1:5000), rabbit anti‐Na^+^‐K^+^‐ATPase α‐1 (06520‐25, 1:1000), and mouse anti‐Tubulin (T6793, 1:1000)). After two washes with PBST, slides were reacted for 2 h at room temperature with Biotinylated Goat anti‐rabbit IgG (ab6720, 1:500) or anti‐mouse IgG (ab6788, 1:500). After incubation for 30 min with ABC mix solution using VECTASTAIN Elite ABC Standard Kit (Vector Laboratories) and washing three washes with PBST, slides were applied with DAB (Dojindo Laboratories) in 8 mM Tris solution until satisfactory coloration was achieved. Treated slides were finally washed with double distilled water and dehydrated through graded ethanol solutions, cleared in xylene, and coverslipped with Malinol (Muto Pure Chemicals).

### 4.5 Scanning Electron Microscopy

5 dpf bichir embryos were fixed in 4% paraformaldehyde in PBS at 4°C overnight and dehydrated in a graded series of methanol. The embryos were critical‐point dried using liquid CO_2_, mounted on a sample holder, covered with gold, and viewed using a Regulus 8100 scanning electron microscope (Hitachi).

### 4.6 Transmission Electron Microscopy

10 dpf bichir embryos were fixed with 2% glutaraldehyde in PBS at 4°C overnight. After rinsing in PBS, samples were postfixed with 1% osmium tetroxide at 4°C for 2 h, dehydrated through a graded ethanol series, and embedded in epoxy resin. Ultrathin sections were cut on an Ultracut‐UCT ultramicrotome (Leica), stained with uranyl acetate and lead citrate, and examined with a JEM‐1400 Plus (JEOL).

### 4.7 Molecular Cloning and Probe Synthesis

Total RNA was extracted from whole embryos of small‐spotted catshark (stage 31), bester (stage 34), bichir (3–10 dpf) and African clawed frog (stage 52), and the gills of adult reedfish and axolotl were transferred to TRIzol (Invitrogen, Thermo Fisher Scientific) and washed with Nucleo Spin RNA XS kit (MACHEREY‐NAGEL). Single‐stranded cDNA was synthesized from total RNA using a Prime Script 1st strand cDNA synthesis kit (Takara Bio) according to the manufacturer's instructions. The gene sequences from the genomic of the animals used in this study were identified using the genome database available at NCBI (https://www.ncbi.nlm.nih.gov/) and the UCSC genome browser (http://genome.ucsc.edu/index.html). cDNA clone of genes was isolated by PCR using forward and reverse primers reported in Supporting Information S1: Table S1. The PCR clone in the pGEM‐T‐Easy Vector (Promega) was used as a template, and a digoxigenin (DIG)‐labeled antisense RNA probe was synthesized by in vitro transcription with T7/SP6 RNA polymerase (Promega) and DIG RNA labeling mix (Roche).

### 4.8 Real‐Time PCR

Total RNA was extracted from gills, skin, heart, guts, liver, kidney, lung, sexual organs, and brain of adult bichir and axolotl were transferred to TRIzol (Invitrogen, Thermo Fisher Scientific) and washed with Nucleo Spin RNA XS kit (MACHEREY‐NAGEL). Single‐stranded cDNA was synthesized from total RNA using a Prime Script 1st strand cDNA synthesis kit (Takara Bio) according to the manufacturer's instructions. Expression levels of *Gcm1* were analyzed by RT‐PCR using this cDNA as a template and specific primers reported in Supporting Information S1: Table S2.

### 4.9 Whole‐Mount and Section *in Situ* Hybridization

All samples for *in situ* hybridization were fixed in 4% paraformaldehyde in 0.01 M PBS at 4°C overnight. Fixed samples were dehydrated with a graded series of methanol. Whole‐mount and section *in situ* hybridization were performed according to a published protocol (Shono et al. 2019).

### 4.10 Animals and Sample Size

Sample sizes varied depending on specimen availability. For the principal embryonic and larval expression analyses, the reported expression patterns were confirmed in at least four individuals of each species. Analyses of adult gills from bichir were based on two individuals, whereas analyses of adult gills from reedfish, bester, bowfin, and spotted gar were each based on a single individual because of limited specimen availability. Representative images are presented throughout the study.

## Author Contributions

Takanori Shono, Norifumi Tatsumi, Tsutomu Miyake and Masataka Okabe conceived and designed the experiments. Takanori Shono and Norifumi Tatsumi performed the experiments. Takanori Shono, Norifumi Tatsumi, Tsutomu Miyake and Shigehiro Kuraku analyzed the data. Takanori Shono, Norifumi Tatsumi, and Masataka Okabe contributed funding. Takanori Shono drafted the manuscript, and all authors contributed to reviewing and editing the final version.

## Conflicts of Interest

The authors declare no conflicts of interest.

## Supporting information


Supporting File

## Data Availability

The data supporting the findings of this study are available within the article and its supporting materials. All data generated or analyzed during this study are included in this published article and its supporting Information files. Publicly available genome assemblies and gene sequences used in the synteny analyses were obtained from NCBI and Ensembl; accession numbers for *Gcm1* and *Gcm2* are listed in Supporting Information S1: Table S1. Primer sequences used for probe synthesis and RT‐PCR are provided in Supporting Information S1: Table S2.
