# Supplementary material for: Two Paralogues as They Like It: Conserved and Divergent Evolution of Vertebrate Gcm Genes
Source: Evol Dev. 2026 Aug 19;28(3):e70057. doi: 10.1111/ede.70057 (PMC13489515; doi:10.1111/ede.70057)
Supplement: Supplementary file 1 [file EDE-28-e70057-s001.docx]

**Electronic supplementary material.**

**This file contains supplementary figures and their legends.**

**Figure S1. Extended synteny of *Gcm2* and *Gcm1* loci in vertebrates.**

The left panel shows the phylogenetic relationships among the examined vertebrate species. The relationships among gnathostome lineages are based on Irisarri et al. (2017). The middle and right panels illustrate extended syntenic regions surrounding the *Gcm2* and *Gcm1* loci, respectively, extending beyond the conserved microsyntenic blocks shown in Figure 1. Despite lineage-specific gene loss or rearrangement, particularly in teleosts and lungfish, the broader genomic context surrounding each locus remains largely conserved among gnathostomes retaining these genes, supporting the inference of orthology based on shared synteny. In cyclostomes, partial conservation of neighboring genes is shown to illustrate probable correspondence to gnathostome *Gcm* loci.

**Figure S2. Syntenic analysis of *Gcm* orthologues in teleost genomes.**

The left panel shows the accepted phylogenetic relationships among the examined species. The middle panel illustrates syntenic regions surrounding *Gcm2*, which is retained in all analyzed teleost genomes. The right panel shows the *Gcm1* locus in spotted gar (*Lepisosteus oculatus*) and the corresponding syntenic regions in teleost genomes, in which *Gcm1* is absent. Despite extensive conservation of neighboring genes, no *Gcm1* orthologue was identified in any teleost genome examined, supporting a lineage-specific loss of *Gcm1* in the common ancestor of teleosts. Teleost species included in the comparison are shown from top to bottom: fugu (*Takifugu rubripes*), medaka (*Oryzias latipes*), zebrafish (*Danio rerio*), channel catfish (*Ictalurus punctatus*), and Asian bonytongue (*Scleropages formosus*). In the phylogenetic tree, spotted gar, a non-teleost actinopterygian, is used as the outgroup.

**Figure S3. Expression of *Gcm1* and *Gcm2* during early embryonic development in bichir.**

(A) RT-PCR analysis of *Gcm1* and *Gcm2* expression in whole embryos from the gastrula stage to 6-day-post-fertilization (dpf), with β-actin used as a loading control. *Gcm2* expression is detectable from the neurula stage, whereas *Gcm1* expression is first observed at 5 dpf, indicating a later onset of *Gcm1* transcription. (B–D) Dorsal views of embryos following whole-mount RNA *in situ* hybridization using *Gcm1* (B) and *Gcm2* (C,D) probes. (B) At 4 dpf, *Gcm1*-expressing cells (arrowhead) are detected at the basal region of the developing external gills (outlined by white dotted lines). (C) At the neurula stage, *Gcm2* expression begins in the emerging external gill primordia (arrowhead). (D) At 3 dpf, *Gcm2* is strongly expressed along the developing external gills (arrowhead). Scale bars: 200 µm (B); 500 µm (C,D).

**Figure S4 Negative** **control images for RNA *in situ* hybridization experiments.**

(A,B) Small-spotted catshark embryos at stage 30 analyzed by whole-mount *in situ* hybridization (WISH) (A) and section *in situ* hybridization (SISH) (B) using a *Gcm1* sense probe as a control.

(C) A bester embryo at stage 34 analyzed by WISH without probes and shown in dorsal view. (D) Horizontal sections of the gill arches of bester larvae at 23-day-post-fertilization (dpf) analyzed by SISH without probes.

(E,F) Bichir embryos at 5 dpf (E) and 10 dpf (F) analyzed by WISH without probes and shown in dorsal view.

(G) Dissected gills of adult bichir analyzed by SISH without probes; arrows indicate vacuole-like structure containing cells (VCCs).

(H,I) Gill sections of adult reedfish analyzed by SISH using a *Gcm1* antisense probe (H) and a *Gcm1* sense probe as a control (I). Arrows indicate *Gcm1*-positive cells, and black dotted lines outline the primary lamellae.

(J,K) Gill sections of axolotl larvae at approximately 5 cm in length analyzed by SISH using a *Gcm1* antisense probe (J,J’) and a *Gcm1* sense probe as a control (K). (J′) Higher-magnification view of the boxed region in (J). Black dotted lines indicate the boundary between the gill epithelium and the underlying mesenchyme. In the antisense section, *Gcm1* expression is localized to the gill epithelium, whereas no specific signal is detected in the corresponding sense control.

(L) African clawed frog embryos at stage 41 analyzed by WISH without probes. Paraffin sections shown in this figure were prepared at a thickness of 14 µm. White dotted lines indicate the gill regions in whole-mount images (A,C,E,F,L).

A, anterior; P, posterior; D, dorsal; V, ventral; pharyngeal arches 1–6 (I–VI); gill arches 1–4 (GA1–GA4); primary lamella (PL); secondary lamellae (SL); epithelium (ep); mesenchyme (m). Scale bars: 1 mm (A, B, C, E, F); 500 µm (L); 200 µm (D); 100 µm (J, K); 50 µm (G, H, I).

**Figure S5. Tissue-specific expression of *Gcm1* in adult bichir and axolotl.**

RT-PCR analysis of *Gcm1* expression in adult tissues from bichir (A) and axolotl (B). Agarose gel electrophoresis of PCR products is shown. cDNA derived from gills, skin, heart, gut, liver, kidney, lung, gonadal tissue, and brain was amplified using *Gcm1*-specific primers, with β-actin used as a loading control. *Gcm1* expression is strongest in the gills, with weaker but detectable signals in several non-pharyngeal tissues. No-template controls are included.

**Figure S6. Histological comparison of the superficial layer of primary gill lamellae tips in adult non-teleost actinopterygians.**

Hematoxylin and eosin–stained sections of the distal tips of primary lamellae from adult gills of spotted gar (*Lepisosteus oculatus*) (A), bowfin (*Amia calva*) (B), bester (*Huso huso × Acipenser ruthenus*) (C) and reedfish (*Erpetoichthys calabaricus*) (D) are shown. (A) Blood vessels (B) are indicated by dotted outlines. (D) In reedfish, vacuole-like structure containing cells (VCCs) characterised by large cytoplasmic vacuole-like structures are indicated by black arrowheads.

Paraffin sections shown in this figure were prepared at a thickness of 14 µm.

Scale bars: 20 µm.

**Figure S7. Comparison of *Gcm1*-expressing cells with ionocyte markers in bichir embryos.**

(A–D) Embryos at 12-days-post-fertilization (dpf) labelled with MitoTracker and observed under light microscopy (A) and fluorescence microscopy (B–D). MitoTracker labels mitochondria-rich cells, including cells of the lateral line and nasal tip (B), ionocytes in the secondary lamellae of external gills (C), and hair cells in the skin surface of embryos labelled with tubulin by immunohistochemistry (D). (E–H) Whole-mount RNA *in situ* hybridization using an Atp1b1 (Na⁺/K⁺-ATPase β1b subunit) probe as a marker of Na⁺/K⁺-ATPase–rich ionocytes (E,F) and a *Gcm1* probe (G,H) in embryos at 5 dpf. Dissected gills (E,G) and yolk-sac membranes (F,H) are shown. Atp1b1-positive cells are restricted to the secondary lamellae (E) and to scattered cells in the dorsal skin (F, dotted outlines), whereas *Gcm1*-positive cells are localized to the basal region of primary lamellae (G, arrowheads) and to scattered cells in the ventral yolk-sac membrane (H, dotted outlines). (I,J) Immunohistochemistry on sagittal sections of embryos at 10 dpf using an antibody against Na⁺/K⁺-ATPase α1 (I) and a negative control without primary antibody (J). Ionocyte markers are not detected in vacuole-like structure–containing cells (VCCs; arrowheads), indicating that *Gcm1*-expressing VCCs are distinct from Na⁺/K⁺-ATPase–positive ionocytes.

Paraffin sections shown in this figure were prepared at a thickness of 14 µm.

Primary lamella (PL); secondary lamellae (SL). Scale bars: 2 mm (A,B); 1 mm (C); 500 µm (E,G); 50 µm (I,J); 20 µm (D); 10 µm (F,H).

**Table S1. Accession numbers of *Gcm* genes used in this study.**

Accession numbers of *Gcm1* and *Gcm2* genes used for synteny analyses presented in Figure 1 and electronic supplementary material Figures S1 and S2.

**Table S2. Primer sequences used in this study.**

Primer sequences used for RT-PCR and RNA *in situ* hybridization.
